# Antibody numbering schemes: advances, comparisons and tools for antibody engineering

**DOI:** 10.1093/protein/gzaf005

**Published:** 2025-04-01

**Authors:** Zirui Zhu, Hossein Ashrafian, Navid Mohammadian Tabrizi, Emily Matas, Louisa Girard, Haowei Ma, Edouard C Nice

**Affiliations:** Department of Chemistry and Biochemistry, The Ohio State University, 151 W. Woodruff Ave. Columbus, OH 43210, United States of America; Chemistry Graduate Program, The Ohio State University, 151 W. Woodruff Ave. Columbus, OH 43210, United States of America; Department of Chemistry and Biochemistry, The Ohio State University, 151 W. Woodruff Ave. Columbus, OH 43210, United States of America; Chemistry Graduate Program, The Ohio State University, 151 W. Woodruff Ave. Columbus, OH 43210, United States of America; Department of Chemistry and Biochemistry, The Ohio State University, 151 W. Woodruff Ave. Columbus, OH 43210, United States of America; Chemistry Graduate Program, The Ohio State University, 151 W. Woodruff Ave. Columbus, OH 43210, United States of America; Department of Chemistry and Biochemistry, The Ohio State University, 151 W. Woodruff Ave. Columbus, OH 43210, United States of America; Department of Chemistry and Biochemistry, The Ohio State University, 151 W. Woodruff Ave. Columbus, OH 43210, United States of America; Department of Mechanical and Aerospace Engineering, Case Western Reserve University, 10900 Euclid Ave. Cleveland, OH 44106, United States of America; Department of Biochemistry and Molecular Biology, Monash University, Wellington Road, Clayton, VIC 3800, Australia

**Keywords:** Antibody, Numbering schemes, Computational tools, Protein engineering, Antibody engineering

## Abstract

The evolution of antibody engineering has significantly enhanced the development of antibody-based therapeutics, enabling the creation of novel antibody formats tailored for specific applications. Since the introduction of the Kabat numbering scheme in 1977, various schemes have been developed and modified, forming the foundation for multiple antibody engineering projects. The tools associated with these schemes further facilitate the engineering process. However, discrepancies among current numbering schemes can lead to confusion. This study examines various numbering schemes and related tools, providing new insights into antibody variable domains. Improved understanding of antibody numbering and related tools holds significant potential for more precise and efficient antibody design, thereby advancing antibody-based therapeutics and diagnostics.

## Graphical Abstract

**Graphical Abstract:**
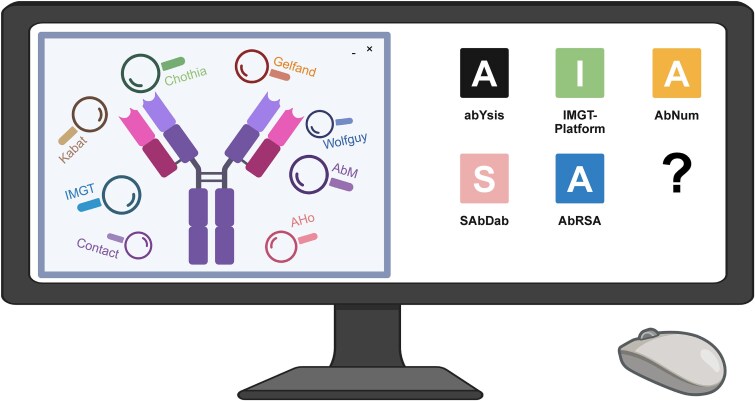

Created with BioRender.com

## Introduction

Antibodies have gained prominence as vital therapeutic agents in recent years, owing to their remarkable efficacy and safety in addressing major diseases such as cancer, immune disorders, infectious diseases, and hematological conditions (Lyu *et al.*, 2022). As of November 24, 2024, data from Umabs-DB reveals that a total of 376 antibody therapies have received approval from at least one regulatory agency worldwide, with approximately half of these in the Immunoglobulin G (IgG) format, while the remainder include antibody fragments and antibody-drug conjugates (Umabs-DB; https://umabs.com, accessed Dec 12^th^ 2024). Antibodies, with their diverse functions such as neutralization, opsonization, complement pathway activation, and antibody-dependent cell-mediated cytotoxicity, have been exploited to develop therapeutics for treating a wide range of diseases, from cancer to immune-related disorders (Sharma *et al.*, 2023).

**Figure 1:**
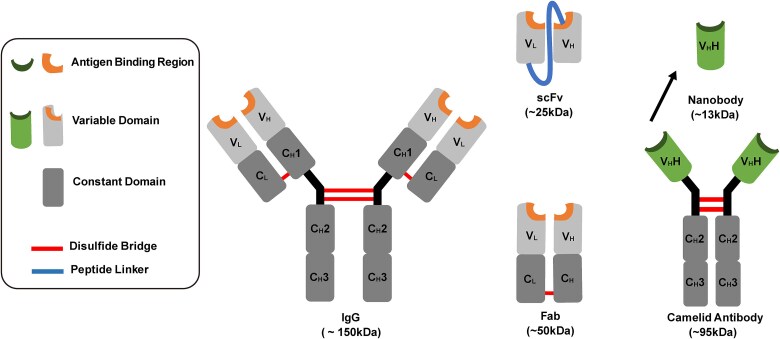
**Structures of antibody and antibody fragments.** The structure of a typical IgG molecule with four polypeptide chains joined by disulfide bonds. The four polypeptide chains consist of two heavy chains (denoted by subscript H) and two light chains (denoted by subscript L). Each heavy chain is composed of a variable domain (VH) bound to three constant domains (CH1, CH2, CH3). Each light chain consists of one variable domain (VL) bound to one constant domain (CL). The Fab fragment is an immunoglobulin fragment composed of one heavy and one light chain, each containing a variable and constant domain. The ScFv fragment contains both the light and heavy chain variable domains joined by a peptide linker. The heavy chain antibody from Camelids is shown with the variable heavy (VHH) single domain antibody (nanobody) denoted above.

Immunoglobulins are Y-shaped glycoproteins (~150 kDa) composed of two heavy chains linked by disulfide bonds and two light chains. Each chain features a variable (V) domain and several constant (C) domains (Fig. 1A). The variable domains, located at the tips of the Y structure, are responsible for antigen binding and consist of two beta sheets, which are stabilized by a conserved disulfide bond (Chothia *et al.*, 1998). These beta sheets are connected by loops, forming a total of six complementarity-determining regions (CDRs) that confer antigen specificity (Chothia *et al.*, 1998; Ofran *et al.*, 2008). The variable domains also include several beta strands that contribute to the structural framework (Chothia *et al.*, 1998; Ofran *et al.*, 2008). In contrast, the constant domains define the antibody’s isotype and mediate effector functions, such as immune cell interactions and complement activation (Sela-Culang *et al.*, 2013).

Advances in antibody engineering have revolutionized the development and application of antibody-based therapeutics, enabling the design of novel antibody formats tailored for specific clinical and research needs (Singh *et al.*, 2023; Ma *et al.*, 2024; Mills *et al.*, 2024), Traditional full-length antibodies, such as IgG, offer robust antigen binding and effector functions but have limitations in tissue penetration and manufacturability for certain applications (Soleimanizadeh *et al.*, 2021; Rodríguez-Nava *et al.*, 2023). Antibody fragments, including Fab fragments, single-chain variable fragments (scFvs), and nanobodies, have been developed to address these challenges (Fig. 1). The Fab fragments retain the antigen-binding capacity of the parent antibody but lack the Fc region, making them smaller and less immunogenic (Rodrigo *et al.*, 2015; Chiu *et al.*, 2019) The scFvs, composed of the variable domains of heavy and light chains connected by a flexible peptide linker, are even more compact and versatile, enabling applications requiring rapid clearance or improved tissue penetration (Monnier *et al.*, 2013; Muñoz-López *et al.*, 2022). Lastly, nanobodies, derived from camelid heavy-chain-only antibodies, are the smallest functional antibody fragments, with remarkable stability, solubility, and the ability to target otherwise inaccessible epitopes (Jin *et al.*, 2023). Engineering techniques such as affinity maturation, humanization, and the creation of bispecific and multispecific molecules have further expanded the utility of antibody fragments (Safdari *et al.*, 2013; Liu *et al.*, 2017; Tabasinezhad *et al.*, 2019; Madsen *et al.*, 2024; Magliery, Thomas *et al.* 2024) These advances allow precise control over binding properties, enhanced pharmacokinetics, and reduced immunogenicity (Safdari *et al.*, 2013; Liu *et al.*, 2017; Tabasinezhad *et al.*, 2019; Madsen *et al.*, 2024). Moreover, antibody engineering has facilitated the incorporation of fragments into multifunctional constructs, such as antibody-drug conjugates and chimeric antigen receptor (CAR) T-cell therapies (Khan *et al.*, 2022; Tsuchikama *et al.*, 2024). Together, these innovations have further broadened the scope of antibody-based solutions, driving progress in diagnostics, targeted drug delivery, and the treatment of complex diseases.

The complementary determining regions (CDRs) within the variable domains of antibodies play a crucial role in their adaptability, underpinning their widespread use in therapeutic, diagnostic, and research applications (Gabrielli *et al.*, 2009; Bhatta and Humphreys, 2018). In contrast, the framework regions (FRs) serve primarily to maintain the structural integrity of the antibody (Chiu *et al.*, 2019). Additionally, insertion sites are frequently situated within the CDRs, where genetic material can be introduced during antibody development, resulting in varying CDR loop lengths that enhance specificity (de Wildt *et al.*, 1999). Defining the CDRs and the insertion sites is an essential first step for downstream antibody engineering. Since the 1970s, when Kabat and Wu introduced the Kabat numbering system based on sequence alignments of 77 Bence-Jones proteins and immunoglobulin light chains, various numbering schemes have emerged in the past 50 years, each adopting different methodologies (Kabat and Wu, 1971). However, aligning these schemes often reveals inconsistencies, which can pose challenges for antibody engineering projects (Dondelinger *et al.*, 2018; Patel *et al.*, 2023). A notable gap exists in the objective evaluation of these differing numbering systems, making it difficult to determine the most suitable approach for specific projects (Dondelinger *et al.*, 2018; Patel *et al.*, 2023). Recently, a statistical analysis of the four major numbering schemes was made, highlighting the need to reassess these frameworks and propose fresh perspectives for improvement (Zhu *et al.*, 2024).

With increasingly precise methods for defining CDRs through various numbering schemes, the advancement of bioinformatic tools and specialized databases has significantly propelled antibody research into a new era (Kiermer, 2008; Dunbar *et al.*, 2014, 2016; Raybould *et al.*, 2020; Abanades *et al.*, 2022). These tailored databases cater to diverse research needs, further enhancing the efficiency and specificity of antibody-related studies. Such modern tools and resources provide valuable support for accelerating ongoing antibody research. This review examines the major existing numbering schemes, as well as the databases and tools developed for antibody numbering.

## Numbering schemes for immunoglobulin variable domains

The objective of numbering schemes is to establish a consistent antibody scaffold that aligns with the target antibody based on information derived from the antibody database. By aligning the amino acid sequence of the target antibody with a specific numbering scheme, it becomes possible to determine a residue’s location within the three-dimensional structure and classify it as part of the framework or CDR regions. This, in turn, aids in identifying critical residues involved in antibody binding and facilitates the delineation of CDRs during the humanization process.

Currently, a range of technologies exists to optimize various biophysical properties of antibodies. One commonly employed technique is affinity maturation, which enhances antibody affinity, avidity, and anti-pathogen activity (Doria-Rose and Joyce, 2015). Another widely used approach is CDR grafting, which reduces immunogenicity and improves the activity of xenogeneic antibodies within the human immune system (Kim and Hong, 2012). The accurate definition of CDRs is vital for the success of these engineering methods. To facilitate this process, numbering schemes have been developed by aligning antibody information stored in database. These numbering schemes leverage the distinct frequencies of amino acids observed in the conserved framework regions and the CDRs of antibodies to define the CDRs (Chothia and Lesk, 1987; Nowak *et al.*, 2016; Krawczyk *et al.*, 2018).

Nowadays, numerous amino acid numbering schemes have been proposed and documented in the scientific literature. It is crucial to systematically summarize the definitions, strengths, and limitations of each scheme to assist researchers in selecting the most appropriate scheme for defining CDRs within their target antibodies. The following section will provide a comprehensive overview and comparison of different antibody numbering schemes.

### Sequence -based numbering schemes

In general, the development of different antibody numbering schemes can be categorized into two approaches: sequence-based and structure-based. The sequence-based approach relies on the analysis of amino acid sequences to determine CDR and FR boundaries. It involves comparing multiple antibody sequences and defining conserved regions that correspond to FRs, while variations in length are accommodated by placing indels (insertion/deletion points) at specific positions. (Kabat and Wu, 1971; Kabat, 1991) This approach primarily utilizes sequence alignment techniques to identify conserved motifs and differentiate between CDRs and FRs. At present, two prominent sequence-based numbering schemes used in antibody research are the Kabat numbering scheme and the IMGT numbering scheme.

#### Kabat numbering scheme

The Kabat numbering scheme is one of the most widely accepted systems for the numbering of amino acid residues in the variable regions of antibodies. The system was first proposed in the 1970s under the methods of Elvin A. Kabat. In 1970, Kabat and Wu aligned 77 Bence-Jones protein sequences, a type of immunoglobulin light chain, with other immunoglobulin light chain sequences to identify regions of highest sequence similarity (Wu and Kabat, 1970). Based on the alignment results, they determined the hypervariable regions of antibodies by dividing the number of different amino acids at a given position by the frequency of the most common amino acids at that position (Kabat and Wu, 1971). Six hypervariable regions characterized by significant variability in amino acid composition were identified through this analysis. These regions were observed within the light chain residues 24–34, 50–56, and 89–97, while within the heavy chain, they were found at residues 31-35b, 50–65, and 95–102 (Kabat and Wu, 1971; Capra and Kehoe, 1974; Kabat, 1991). Additionally, the alignment revealed variations in the length of these hypervariable regions, indicating structural flexibility and diversity within specific regions of the antibody chains (Wu and Kabat, 1970; Capra and Kehoe, 1974). These CDRs were then labeled as CDR L1,2,3 and H1,2,3 respectively.

The Kabat numbering scheme was developed at a time when immunoglobulin structural information was limited or unavailable; therefore, it is primarily based on the sequential order of amino acids. The Kabat scheme assigns labels to residues in a successive manner, considering the most common sequence lengths observed in antibodies. Insertions are denoted by letters in the numbering scheme (*e.g.* 27a) (Chiu *et al.*, 2019). The Kabat numbering scheme primarily places insertion sites within the middle or at the end of the CDRs. For example, in the light chain, insertions such as 27a-f can occur in LCDR1, 95a-f in LCDR3, and 100a-k in HCDR3. However, it is worth noting that some insertion sites are also found within the FR, such as 106a in L-FR4 and 82a-c in H-FR3 (Kabat, 1991). Interestingly, according to the Kabat numbering scheme, no insertion sites are defined within LCDR2 (Kabat, 1991). Many of the CDRs as defined by Kabat have strict lengths and more definitive residue lengths. Opposingly, HCDR3, the region most highly involved in antibody binding, has significant variability in the number of residues involved (length varies from 2–23 residues) (Martin, 2010).

The Kabat numbering scheme, which was the first systematic method to delineate the CDRs based on sequence alignment, continues to serve as the standard for antibody sequence numbering in diverse systems. As the amount of antibody sequence data has significantly increased through published research, various databases were established primarily based on the Kabat numbering scheme to aid researchers in gaining a deeper understanding of antibody protein science. Prominent among these databases are the Kabatman, Kabat, and in part the abYsis database. (Martin, 1996; Johnson and Wu, 2000; Swindells *et al.*, 2017). These databases serve as valuable resources for storing, organizing, and retrieving antibody sequence information from a wide range of sources.

**Figure 2:**
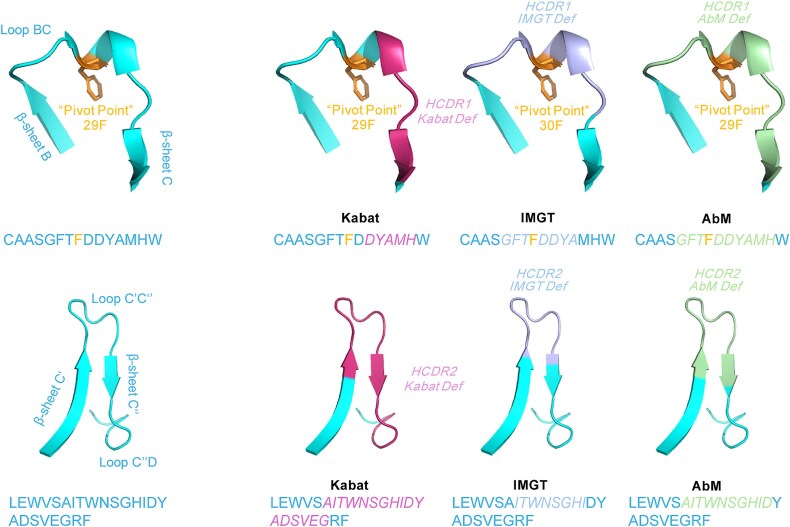
**The structural alignments of the CDRs based on different numbering schemes. (Upper)** The structure of the HCDR1 loop of a Fab structure (PDB: 4NYL) defined based on the Kabat, IMGT and AbM numbering schemes respectively. **(Lower)** The structure of the HCDR2 loop of a Fab structure (PDB: 4NYL) defined based on the Kabat, IMGT and AbM. The framework is shown in one representation, and the pivot point in the CDR1 loop is indicated. The HCDR loops are depicted according to the respective numbering schemes. Amino acids are represented using the one-letter code, and the CDRs are highlighted in *italic*. The figure was generated using the pymol software (PyMOL™ 2.5.5) (Schrödinger, 2024).

Since the system was built on the most common sequence lengths and only a limited number of sequences the scheme does not match many 3D structures (Dondelinger *et al.*, 2018). Sequences that contain uncommon indels were not included in the Kabat scheme and certain defined insertion points in LCDR1 and HCDR1 do not fit with their position in the 3-dimnesional structure (Dondelinger *et al.*, 2018). This is one of the downsides of the Kabat scheme as it sometimes does not accurately represent the CDR in 3D space. Previous analyses have also indicated that approximately 10% of the entries annotated using the Kabat numbering scheme exhibit errors or inconsistencies (Abhinandan and Martin, 2008). It has been observed that the HCDR1 region as defined by the Kabat numbering scheme often exhibits significant discrepancies when compared to other numbering schemes such as Chothia, Martin, and IMGT. This discrepancy may arise from differences in the definition and alignment of the HCDR1 region between these schemes. Due to the fact that the Kabat numbering scheme primarily relies on the frequencies of AAs observed in antibody sequences, this approach can result in the definition of wider CDRs compared to other numbering schemes. Finally, the recent statistical analysis revealed inaccuracies in the Kabat numbering scheme for defining HCDR1 and HCDR2 (Fig. 2). In that study, the pivot point was identified as a conserved residue (L29 in kappa, L30 in lambda, H29 in variable heavy, Kabat numbering) that segments CDR1 into two subloops and anchors its conformation. Specifically, HCDR2 was found to overestimate its range by including additional beta sheets and loops, while HCDR1 excluded the N-terminal loop, deviating from the conserved ‘M-shaped’ CDR1 loop structure. (Zhu *et al.*, 2024).

#### IMGT numbering scheme

Marie-Paule Lefranc introduced the IMGT unique numbering system with the aim of simplifying the comparison of immunoglobulin (IG) and T cell receptor (TR) sequences, as well as major histocompatibility complex (MHC) and related proteins of the immune system (RPI) sequences, across human and other vertebrate species (Lefranc, 1997; Lefranc *et al.*, 1999). Originally, the generation of the unique numbering scheme for IMGT was based on the significant conservation of structure of the variable region. (Lefranc, 1997). Later, the IMGT numbering scheme annotation was extended to the whole variable domain by rearranging the IG and TR V-GENEs and V–D–J-GENEs which comprises position 105–117 (corresponding to CDR3-IMGT) and position 118–129 (corresponding to FR4-IMGT). (Lefranc *et al.*, 2003).

Unlike other numbering schemes, the IMGT numbering method adopts a continuous counting system from 1 to 129, which is derived from the alignment of germ-line V sequences (Lefranc *et al.*, 2003). To maintain consistency, the IMGT numbering system generally avoids the use of insertion sites. However, an exception is made for the insertion between positions 111 and 112 specifically for proteins with a HCDR3 longer than 13 amino acids. Additionally, if a residue is absent in a particular sequence, no number is assigned to it (Lefranc *et al.*, 2003). This approach ensures that the numbering remains consistent and does not introduce artificial placeholders for absent or inserted residues.

Thanks to the unique numbering system utilized in IMGT, conserved amino acids are consistently found at the same positions. For example, C23, W41, L89, and C104 retain their fixed locations in the numbering scheme (Kabat, 1991). This feature greatly simplifies sequence alignments, as researchers can easily identify and compare these conserved residues across different sequences. The consistent positioning of these amino acids aids in recognizing structural and functional motifs, enhancing the understanding of sequence similarities and differences within the aligned sequences. Furthermore, the database utilized for the IMGT alignment encompasses not only antibody genes but also the entire immunoglobulin superfamily. This broad inclusion of genes expands the applications of the IMGT unique numbering system to other antibody scaffolds and related proteins. Lastly, based on the IMGT numbering scheme, various applications have been developed including the online database and alignment tool IMGT/DomainGapAlign (Ehrenmann *et al.*, 2010; Ehrenmann and Lefranc, 2012) sequence analysis software and server IMGT/V-QUEST (Brochet *et al.*, 2008)^.^ and 2D visualization tool IMGT/Collier-de-Perles (Lefranc, 2011).

While the IMGT unique numbering scheme avoids the ambiguity of insertion sites found in other numbering schemes such as Kabat and Chothia, its application to engineered antibodies with non-standard length CDRs is somewhat limited. This is because the IMGT scheme defines the longest amino acid sequences as the reference, which may not account for antibodies with atypical CDR lengths. Unlike the Kabat and Chothia numbering schemes, which utilize insertion letters to indicate the presence of insertions at specific positions, the IMGT numbering scheme requires a comprehensive scan of the entire antibody sequence to identify any missing numbers which can sometimes be a worse visual indicator. Moreover, the initial IMGT numbering scheme frequently introduced insertion sites at the end of the CDRs, which did not consistently align with the antibody’s true structural locations. However, this concern has been successfully addressed through the implementation of the IMGT/V-QUEST sequence analysis software. (Brochet *et al.*, 2008). IMGT/V-QUEST resolves this issue by strategically positioning insertions within the middle of the CDRs, resulting in a more precise alignment that appropriately reflects the antibody’s structural context (Brochet *et al.*, 2008).

**Figure 3:**
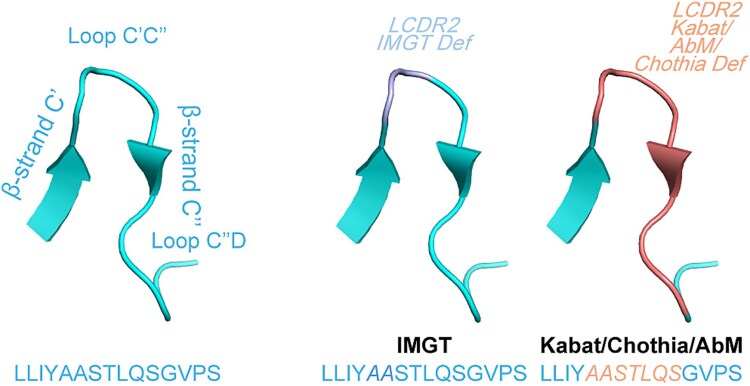
**The structural alignments of the LCDR2 defined by different numbering schemes.** The structure of the LCDR2 loop of a Fab structure (PDB: 4NYL) defined based on the IMGT, Kabat, Chothia and AbM numbering scheme respectively. The framework and HCDR loops are visually distinguished in the structure: HCDRs defined by IMGT are styled differently from those defined by Kabat, Chothia, and AbM to highlight differences in loop definition. Amino acids are shown in one-letter code, and CDRs are emphasized in *italic*. The figure was generated using the pymol software (PyMOL™ 2.5.5) (by Schrödinger, 2024).

The structural analysis shows that the IMGT numbering scheme can accurately define the beginning and end of a CDR, with only minor differences in the loop margins when compared to other numbering schemes (Zhu *et al.*, 2024). Notably, the IMGT scheme defines the LCDR2 loop within a more constrained spatial region, potentially missing key residues (Fig. 3). In contrast, other major numbering schemes (Kabat, AbM, Chothia) tend to overestimate the LCDR2 loop range. Furthermore, the IMGT numbering includes two relatively conserved residues at positions 105 and 106, compared to the other HCDR3 residues, leading to a higher overall conservation level than observed with other numbering schemes (Table 1).

**Table 1:** The amino acid distribution at residues of interest in the variable heavy domain. The amino acid distribution of the H102 – H108 based on Kabat numbering scheme was obtained from abYsis (http://www.abysis.org/abysis/, accessed Dec 12^th^ 2024) and their CDR definition based on the IMGT, Kabat, and AbM numbering schemes. Amino acids are represented in one-letter code and only the amino acids with >4% distribution was presented in the table. The table was adapted from (Zhu *et al.*, 2024).

**HFR3**						**HFR3 (Kabat/AbM) HCDR3 (IMGT)**				**HCDR3**			
**H102**		**H103**		**H104**		**H105**		**H106**		**H107**		**H108**	
Y	99%	Y	86%	C	100%	A	85%	R	70%	G	18%	G	16%
		F	13%			T	6%	S	4%	D	18%	R	10%
						V	5%	T	4%	Y	8%	Y	10%
										E	7%	S	7%
										S	7%	P	7%
										R	6%	L	7%
										A	5%	D	6%
										V	5%	A	5%
										L	4%	T	4%
												V	4%
												K	4%

### Structure-based numbering scheme

Recently, the structure-based approach to antibody numbering schemes is gaining prominence in antibody research. With the increasing availability of 3D antibody structures determined through techniques like X-ray crystallography, NMR, and Cryo-EM, scientists now have a valuable resource for accurately defining CDRs based on structural information. The structure-based numbering schemes rely on the analysis of variable loop structures within the antibody to determine the boundaries of CDRs and FRs. The notable examples of structure-based numbering schemes include the Chothia numbering scheme, the Contact numbering scheme, the Martin numbering scheme, the Gelfand numbering scheme and the Honneger’s numbering scheme (AHo’s).

#### Chothia numbering scheme

After the Kabat numbering scheme reported by Kabat and Wu in 1971, the understanding of antibody structure and the distinction between hypervariable regions (CDRs) and more conserved regions (Framework regions) began to develop. However, in 1979, Padalan observed that certain residues classified as conserved in the FRs according to the Kabat scheme were also involved in forming the hypervariable loops in the 3D structure of antibodies (Padlan, 1994). At that time, several structural studies provided compelling evidence that despite differences in sequence, hypervariable loops of the same size can adopt identical conformations. (Segal *et al.*, 1974; Saul *et al.*, 1978; Marquart *et al.*, 1980). These observations suggest that the conformation of the hypervariable loop is also determined by its structural context and the interactions it forms within the antibody structure rather than solely relying on the specific amino acid sequence.

In 1987, Cyrus Chothia and Arthur M. Lesk observed that certain sets of residues within the immunoglobulin structure were responsible for adopting specific hypervariable conformations which they referred to as ‘canonical structures.’ The other residues in the hypervariable loops that were not directly involved in the canonical structures played a role in modulating the surface presentation of the loops to antigens. (Chothia and Lesk, 1987) Based on their extensive analysis of the hypervariable domains (except HCDR3) in the high-resolution immunoglobulin structures, Chothia and Lesk identified 18 ‘standard’ canonical structures that exhibit similarities in the conformation of most of the hypervariable domains tested which suggests high conservation of the hypervariable loop structures in immunoglobulins (Al-Lazikani *et al.*, 1997).

Based on their observation, they introduced a novel numbering scheme for antibodies based on their structural analysis called the Chothia numbering scheme. The Chothia numbering scheme, like the Kabat scheme, does have notable differences, particularly in the definition of certain hypervariable loops and the placement of insertions in certain regions. The Chothia numbering scheme defines the six hypervariable regions in antibody sequences based on their residue positions: LCDR1 (24–34), LCDR2 (50–56), LCDR3 (89–97), HCDR1 (26–35), HCDR2 (52–56), and HCDR3 (95–102). And the hypervariable loops are defined by their 3D location outside the beta sheet structure, which is determined by superimposing available crystal structures, to generate a conserved beta sheet framework. (Chothia and Lesk, 1987). In the Chothia numbering scheme, certain insertion sites have been shifted compared to the Kabat numbering scheme to make the insertion site structurally correct. For example, in the LCDR1, the insertion site has been shifted from L27 to L30. Similarly, in the HCDR1, the insertion site has been shifted from H35 to H31. It is worth mentioning that in the Chothia numbering scheme, the original insertion sites in LCDR1 and LCDR3 were defined as L31 and L93 respectively. (Chothia and Lesk, 1987; Chothia *et al.*, 1989). In their latest publication, the Chothia numbering scheme corrected the insertion sites in LCDR to L30 and L95, which better align with the corresponding structural positions in the antibody. (Al-Lazikani *et al.*, 1997).

Overall, the Chothia numbering scheme helped to better define the structural loops of antibodies by aligning crystal structures of the variable regions to better fit the spatial arrangement of residues. (Chothia and Lesk, 1987). From the initial set of 10 canonical structures proposed by Chothia in 1987, the number of identified canonical structures has expanded to 26 as of 2016 (Chothia and Lesk, 1987; Nowak *et al.*, 2016; Wong *et al.*, 2019a). With the continuous accumulation of structural data, our knowledge of CDR loop conformations and variability has significantly evolved. Several useful tools, such as SCALOP (Wong *et al.*, 2019a) and abYsis, have been developed based on the identified canonical structures, playing a crucial role in enhancing our understanding of CDR structures.

**Figure 4:**
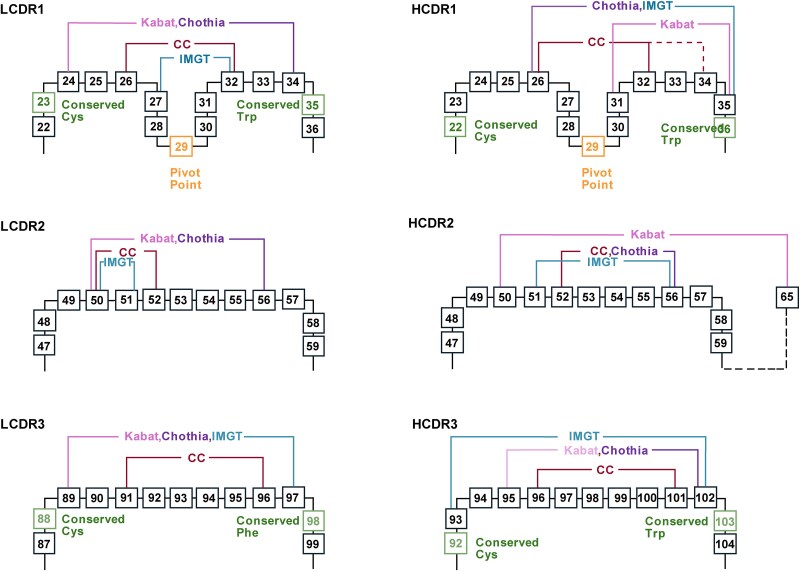
**A representation of the CDR definition based on different numbering schemes.** The 2D map of the residues in the CDR loops are defined based on the IMGT, Kabat, Chothia, and Consensus Chothia (CC) numbering schemes respectively. The number in the box represents the residue number based on the Kabat numbering schemes. Note the dashed line at the end of the Chothia CDR-H1 loop, where the Kabat numbering scheme places the insertion site at H35A and H35B, causing variation between H32 and H34 depending on loop length. Several conserved residues (distribution >99%) and featured residue (pivot point in CDR1) were highlighted. The different CDR loop definitions and key residues are visually distinguished using unique styles or labels corresponding to each scheme or feature.

The main limitation of the Chothia numbering scheme lies in its limited use compared to the more widely accepted Kabat numbering scheme. This inconsistency leads to confusion when comparing CDRs annotated using these two different systems. Another notable concern is because various publications from Chothia appear to present somewhat different definitions of the CDRs, particularly when considering both the figures and the text, making it challenging to consistently define CDRs based on the Chothia scheme. Martin’s group has compiled and summarized these definitions from past literature, referring to it as the ‘consensus interpretation of Chothia’ (Zhu *et al.*, 2024). However, this consensus definition has not been adopted by commonly used antibody annotation servers such as abYsis. The Chothia scheme used in abYsis closely resembles the Kabat numbering scheme with the exception of the tailored regions in HCDR1 and HCDR2 (Fig. 4). While the ‘consensus’ Chothia definition appears to offer a more conservative approach for defining the CDRs, it interestingly aligns more closely with the IMGT numbering scheme in some loops. Additionally, similar to the Kabat scheme, the Chothia numbering relies on the most frequently observed CDR lengths, which may not accurately capture antibodies with atypical CDR length.

#### Martin numbering scheme.

In a study conducted by K.R. Abhinandan and Andrew C.R. Martin in 2008, the performance of the Kabat numbering scheme was evaluated using their program, AbNum (Abhinandan and Martin, 2008). During the numbering process, they discovered an error in the Kabat scheme where a group of antibodies with three residues inserted at H52 (in HCDR2) was mistakenly labeled as inserted at H82 (in HFR3). This mistaken annotation led to an inaccurate depiction of the end of HCDR2 (Abhinandan and Martin, 2008). Moreover, when Cyrus Chothia and Arthur M. Lesk proposed the Chothia numbering scheme in 1987, they corrected the incorrect inserted sites in the CDRs but not the frameworks. All these observations suggest additional corrections should be made to introduce the insertion sites at the structurally correct positions.

After a careful structural analysis of the antibody structures extracted from the SACS database, Martin and his colleague noticed that H82 is unlikely to accommodate insertions as the structure was relatively buried in the beta sheet (Allcorn and Martin, 2002; Abhinandan and Martin, 2008). Further sequence alignments suggests that H72 is likely the insertion site of the three residues (Abhinandan and Martin, 2008). They also validated the correct insertion site at L52 in LCDR2, which was not specifically addressed in the Chothia numbering scheme since the examined structures exhibited the same length for the LCDR2 loop. Additionally, during the numbering process using the AbNum program, they identified a novel insertion/deletion position at position H6 in the heavy chain, which was subsequently adjusted to H8 in the Martin numbering scheme (MacCallum *et al.*, 1996; Abhinandan and Martin, 2008).

**Figure 5:**
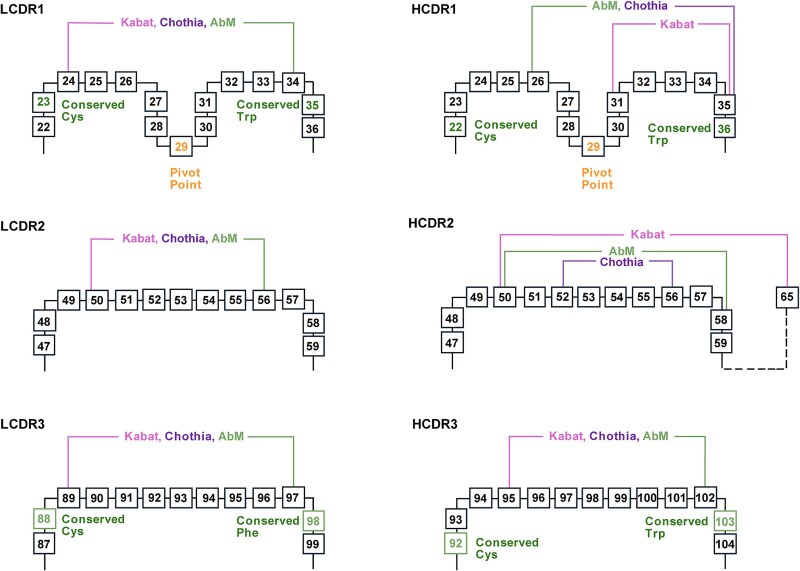
**A representation of the CDR definition based on different numbering schemes.** The 2D map of the residues in the CDR loops are defined based on the Kabat, Chothia, and AbM numbering scheme respectively. The number in the box represents the residue number based on the Kabat numbering schemes. Several key residues are emphasized: highly conserved residues (present in more than 99% of sequences) and a pivot point residue in CDR1. Distinct visual styles or labeling are used to differentiate between CDR loops defined by each numbering scheme and to highlight conserved and functionally relevant residues.

The Martin numbering scheme, often referred to as an ‘enhanced’ Chothia definition or the ‘AbM’ scheme, is a more advanced structure-based approach. Building upon the original Chothia scheme, Martin introduces various corrections and refinements based on detailed structural analyses and comparisons with other numbering systems. Structurally, the Martin scheme adopts the same definitions of the CDRs as the Kabat and Chothia schemes but includes modifications to HCDR1 and HCDR2, making it more structurally accurate (Fig. 5). This includes extending the N-terminal loop beyond the pivot point in HCDR1 and removing incorrectly defined residues in HCDR2 according to the Kabat scheme. By including additional residues at the boundaries of the CDR loops, the Martin scheme also increases the average conservation level in statistical analysis. Overall, the Martin numbering scheme offers the most comprehensive definition of CDRs, correcting several critical discrepancies to ensure structural accuracy.

#### Gelfand numbering scheme

In 1995, Israel M. Gelfand and Alexander E. Kister introduced a novel numbering scheme called the Gelfand numbering scheme. This scheme was developed through a combination of structural and statistical analyses to investigate the relationship between sequence, secondary structure, and three-dimensional structure of antibodies (Gelfand and Kister, 1995). The Gelfand numbering scheme originated from the statistical analysis of predicted structures based on the sequences derived from the Kabat database. During this analysis, Gelfand and Kister identified specific positions within the antibody strands and loops that displayed a high degree of conservation or shared common features, such as hydrophobic properties, across a wide range of antibody sequences (Gelfand and Kister, 1995). Later on, Gelfand and Kister extended their analysis by constructing invariant coordinates based on the two-fold symmetry and calculating the weighted center of mass for the variable domains (Gelfand *et al.*, 1996, 1998). Subsequent structural analysis revealed that 73 out of 96 positions tested in the light chain and 72 out of 87 positions tested in the heavy chain satisfied the alpha carbon distance criterion calculated based on their conservative coordinates. (Gelfand *et al.*, 1996) Furthermore, Gelfand and Kister developed an algorithm based on the consistent distances between C_ɑ_ atoms across different immunoglobulin variable domains. This algorithm was designed to identify and define the geometric core of the protein structure. (Gelfand *et al.*, 1998a; Stoyanov *et al.*, 2000).

**Figure 6:**
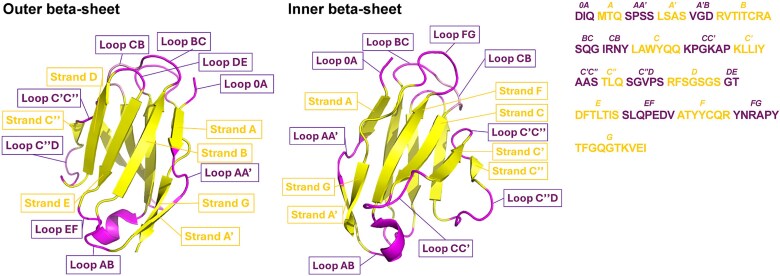
**Structure of the Variable Light Domain Based on the Gelfand Numbering Scheme.** The structure of the variable light domain from a Fab (PDB: 4NYL) is shown, with the domain defined according to the Gelfand numbering scheme. Two structural perspectives are shown: the outer β-sheet (**Left**) and the inner β-sheet (**Middle**). The accompanying amino acid sequence is shown alongside a schematic representation of secondary structure elements (**Right**), distinguishing loops and β-strands based on the Gelfand scheme. These structural elements are differentiated using distinct visual styles or labels. Amino acids are presented using their one-letter codes. The figure was generated using the pymol software (PyMOL™ 2.5.5) (Schrödinger, 2024).

In the Gelfand numbering scheme, an interesting feature is the division of the variable domains based on their secondary structures. The scheme assigns specific names to the 10 strands within each domain, namely A, A’, B, C, C′, C″, D, E, F, and G. (Gelfand and Kister, 1995). Similarly, the 8 loops connecting these strands are named based on the two strands they connect. Thus, the loops are designated as AA’, A’B, CC’, C’C″, C″D, DE, EF, and FG. (Gelfand and Kister, 1995) (Fig. 6). To accommodate the structural arrangement, the first residue that does not belong to the A strand is labeled as 0A. Additionally, the connection loop between strands B and C, which forms an “M”-like conformation with one residue dipped into the structure, is split into two separate loops referred to as BC and CB, respectively. (Chothia and Lesk, 1986; Gelfand and Kister, 1995; Gelfand *et al.*, 1998).

The Gelfand numbering scheme takes a distinct approach by focusing on the conservation characteristics of specific residues and their correlation with secondary structures, rather than solely defining the CDRs and FRs. Instead, it recognizes the significance of conserved residues and their role in upholding the structural integrity and functionality of antibodies. Their findings further supported the notion that specific positions within the antibody structure exhibit conserved characteristics; however, it is worth noting that no further studies or developments related to the Gelfand numbering scheme have been reported since 2009. As a result, there are currently no available tools or resources specifically designed for annotating antibodies using the Gelfand numbering scheme.

#### Honneger’s numbering scheme (AHo’s)

In 2001, Annemarie Honegger and Andreas Plückthun introduced a residue labeling scheme known as Honegger’s numbering scheme (AHo’s), which aimed to preserve positional information derived from the comparison of experimental structures and models. (Honegger and Plückthun, 2001a). The scheme aims to maintain structural integrity by aligning the Cɑ positions within the structurally most conserved core region of the antibody domains. The scheme effectively identifies gap locations suitable for accommodating insertions and deletions by minimizing the average deviation from the averaged structure of the aligned domains.

The Honegger numbering scheme deviates from the unidirectional insertion approach adopted by many other numbering schemes. Instead, it employs a unique strategy of symmetrically inserting and deleting residues at loop or turn regions of the antibody structure. This symmetric placement is carefully determined based on structural criteria to maintain the integrity and stability of the surrounding structure. Unlike other schemes that use alphabetical modifiers to indicate insertions, the Honegger numbering scheme allows for gaps in the numbering system to accommodate the natural length variations observed in germline sequences.

During the structure alignment process, the Honegger numbering scheme identifies specific residues within the domain that constitute the most conserved core region, including residues 3–7, 20–24, 41–47, 51–57, 78–82, 89–93, 102–108, 138–144, as well as highly conserved residues across different species such as C23, W43, C106, and G142. Additionally, the scheme identifies several gaps in the hypervariable regions and the frameworks, including a gap at position 8 in FR1 (∆8), gaps at positions 28 (∆28) and 36 (∆36) in CDR1, gaps at position 63 (∆63) and positions 74–75(∆74,75) in CDR2, gaps at 85–86 (∆85,86) in FR3, and a gap at position 123 (∆123) in CDR3.

Some of the gaps introduced by the Honegger numbering scheme serve to accommodate the length variations observed in variable domains across different proteins within the immunoglobulin superfamily. For example, the ∆8 gap is specifically designed to account for the one-amino acid insertion in VL kappa compared to V_L_ lambda and V_H_, as supported by structural analysis. Similarly, the ∆74,75 and ∆85,86 gaps are strategically placed to accommodate the C-terminal branch being retracted observed in alpha T-cell receptors CDR2 loop as well as the one-residue gap in the beta T-cell receptor at position B86.

In the hypervariable regions, the gaps are carefully designed to align more accurately with the 3D structures of antibodies. For instance, CDR1 plays a crucial role in connecting the two beta-sheet structures in antibodies and therefore exhibits a unique ‘two span bridge’ structural characteristic (Honegger and Plückthun, 2001a). Within the CDR1 segment, a hydrophobic residue (#31 in the Honegger numbering scheme) is typically positioned between the two beta sheets, dividing CDR1 into two distinct loops. The lengths of these two loops show some variations, necessitating the inclusion of two gaps (∆28 and ∆36) in CDR1 to maintain structural alignment. The gap in the CDR 2 has been placed to be centered on position 63 to accommodate the variation of the length of CDR2. The ∆123 position has been added to symmetrically divide CDR3 between the highly conserved C106 and G140 leaving ample space for the diversified CDR3 sequences.

**Figure 7:**
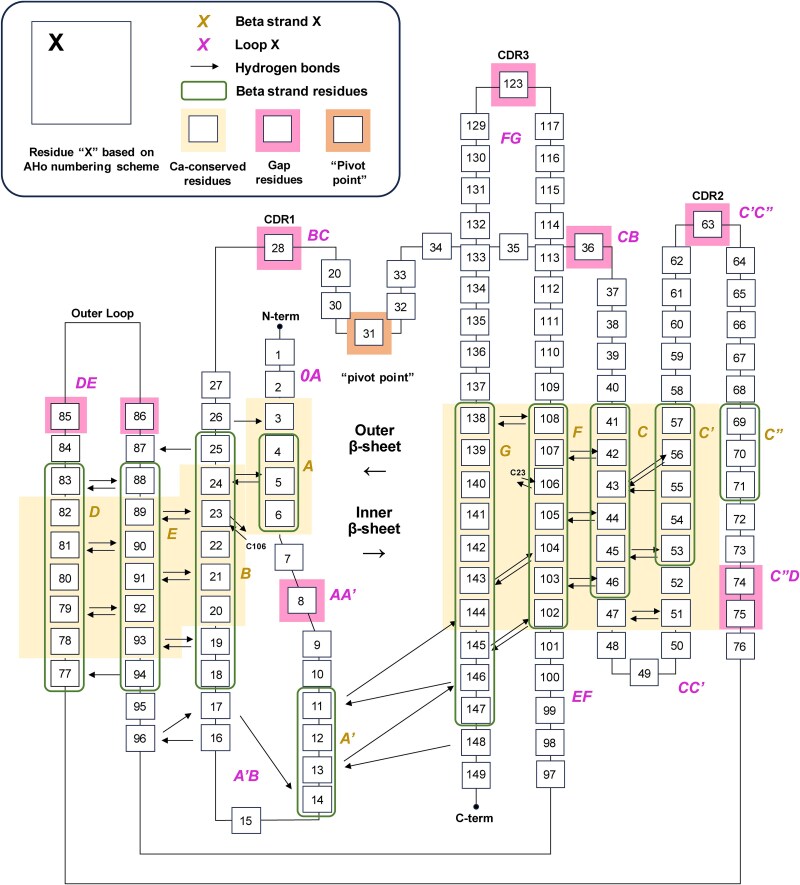
**Consensus Structure and Main-Chain Hydrogen Bonding Pattern of Immunoglobulin Variable Domains.** Residues are numbered according to the AHo numbering scheme. Arrows indicate hydrogen bonds that are conserved across the majority of immunoglobulin variable domain structures. Regions corresponding to loops and turns—typically accommodating sequence gaps—are visually distinguished. Residues whose Cα positions were used for least-squares structural superposition are marked using a distinct visual style. Secondary structure elements are annotated according to the Gelfand numbering scheme. β-strand residues are enclosed in stylized boxes, and each strand is labeled accordingly. Loops and turns are also labeled with unique identifiers to distinguish them from the β-strand regions.The figure was adapted from (Honegger and Plückthun, 2001a; Zhu *et al.*, 2024).

The Honegger numbering scheme offers two significant advantages. Firstly, it takes into account the structural alignment of antibody domains and ensures a better match between the numbering scheme and the actual antibody 3D structures. This results in a more accurate representation of the sequence in relation to the structural features. Secondly, the Honegger numbering scheme provides a unified numbering system for the superfamily of immunoglobulin variable domains. It covers not only the immunoglobulin light chain lambda and kappa variable domains, and the heavy chain variable domain, but also the variable domains of T-cell receptors, including alpha, beta, gamma, and delta. This comprehensive approach allows for consistent and standardized annotation across different variable domain types, facilitating comparative studies and analysis. The AHo numbering scheme and the 2-dimnesional map pioneers a perspective that shifts focus from amino acid sequences to the 2D spatial distribution of the antibody variable domain. This scheme identifies multiple residues with key structural features, and its novel concept of unidirectional insertion represents a breakthrough in the field. When combined with the Gelfand numbering scheme, it effectively illustrates the secondary structure distribution of the antibody and displays the hydrogen bonding relationships between residues (Fig. 7). However, due to the unique numbering approaches used by Honegger, researchers need to be mindful of these differences when comparing data or conducting cross-referencing with studies that employ alternative numbering. Furthermore, like IMGT, the Honegger numbering scheme exhibits limited flexibility, particularly when it comes to accommodating antibodies with longer insertion regions.

#### Contact numbering scheme.

In 1996, Robert M. MacCallum and colleagues introduced a new approach to define the CDRs by analyzing protein structures in the PDB and identifying the specific residues that interact with antigens. Their findings revealed that the residues located at the center of the CDRs, as defined by the Chothia and Kabat numbering schemes, are frequently involved in direct interactions with antigens. In contrast, the residues positioned away from the center of the CDRs primarily interact with larger antigens, while the non-contacting residues within the CDRs contribute to defining the canonical conformations of the CDR loops. Through a thorough analysis of complex structures, the researchers introduced a revised definition of the CDRs based on the Kabat and Chothia numbering scheme. In this new definition, they excluded non-contacting residues that solely contributed to loop conformation and included additional contacting residues not covered by the Kabat and Chothia numbering scheme. Furthermore, they observed that within each CDR, especially LCDR3 and HCDR3, a higher number of contacts were made at the loop’s end closer to the center of the combining site, while the residues in the apical loop region had fewer interactions with the antigen.

Notably, they also observed that the size of the antigen influences the conformation of the CDRs. Through an analysis of antibody surface topology patterns, they identified four subclasses of antibody CDR conformations: concaves and half concaves, predominantly observed in hapten binders; ridged, primarily found in peptide binders; and planar, mostly seen in protein binders.

The Contact Analysis provides us with more insight into the antibody and antigen interactions and their effect on the CDR definition, but the amount of the complex structure examined in this work is relatively limited due to the low amount of available antibody–antigen complex structure at that time. Also, excluding the residues that play a structural role and adding the framework residues that are in contact with the antigen leads to the CDR defined by the Contact Numbering Scheme and shows lower agreement when compared to the other numbering scheme. Also, no information about the insertion sites is mentioned in this work which may limit its use in antibodies with variable length CDRs.

#### Wolfguy numbering scheme

The Wolfguy numbering scheme, introduced by Alexander Bujotzek et al. in 2015, presents an intriguing structural-based approach. It defines the CDRs as a combined definition from the Kabat and Chothia schemes; however, the annotation significantly deviates from other numbering schemes (Bujotzek *et al.*, 2015).

In the Wolfguy scheme, the numbering of the two chain types is not equivalent, and different regions of the domain are denoted by distinct number ranges. The ample number range allocated to the CDRs implies that no insertion site is required in this scheme. Additionally, CDR loop numbers are assigned based on their length and canonical structure, and the index of a CDR position indicates whether the residue belongs to the ascending or descending loop. This unique numbering scheme allows for easy assignment of residues with similar spatial structures using the same number. The spatial conservation of residues assigned with the same number in the Wolfguy scheme provides additional benefits for tasks such as homology modeling; however, due to the highly unique numbering assigned to residues in the Wolfguy scheme, it can sometimes lead to confusion. Comparing the numbering across different schemes can also be challenging due to the distinct nature of the Wolfguy numbering.

### Comparison of currently available numbering schemes

Multiple numbering schemes have been developed over time, and numerous corrections have been implemented to enhance accuracy in defining CDRs. As a result, the technology behind numbering schemes has reached a high level of maturity. However, it is important to note that there is not always a strong consensus among different numbering schemes when applied to the same amino acid sequence input (Table 2). There can be variations and discrepancies between the results obtained from different schemes. Some studies indicated that grafting the combined CDRs defined by multiple numbering schemes may be beneficial (Zhang and Ho, 2017). However, grafting longer CDRs may disrupt the overall stability and requires a stable human antibody framework (Honegger *et al.*, 2009; Kügler *et al.*, 2009).

**Table 2:** Comparison of CDR definitions across different numbering schemes. CDRs were defined using the Kabat, Chothia, Consensus Chothia, AbM, and IMGT methods. Residues in the Kabat, Chothia, Consensus Chothia, and AbM schemes were numbered according to the Kabat system, while IMGT follows its own numbering scheme. Insertion sites were identified and adapted from the abYsis database.

**CDR definition**	**Kabat**	**Chothia**	**Consensus** **Chothia**	**AbM**	**IMGT**
LCDR1	L24-L34	L24-L34	L26-L32	L24-L34	27–38
LCDR2	L50-L56	L50-L56	L50-L52	L50-L56	56–65
LCDR3	L89-L97	L89-L97	L91-L96	L89-L97	105–117
HCDR1	H31-H35	H26-H35	H26-H32	H26-H35	27–38
HCDR2	H50-H65	H52-H56	H52-H56	H50-H58	56–65
HCDR3	H95-H102	H95-H102	H96-H101	H95-H102	105–117
**Insertion Sites**	**Kabat**	**Chothia**		**AbM**	**IMGT**
LFR1					
LCDR1	L27 A-F	L30 A-F		L30 A-F	30–35
LFR2	L39 A	L39 A		L40 A	46.1
LCDR2	L54 A-E	L54 A-E		L52 A-E	57–64
LFR3	L66 A-H	L66 A-H		L68 A-H	84.1–84.6
LCDR3	L95 A-F	L95 A-F		L95 A-F	111.1–112.1
LFR4	L106 A	L106 A		L106 A	127
HFR1	H6 A-D	H6 A-D		H7 A-D	10, 10.1–10.3
HCDR1	H35 A-H	H31 A-H		H31 A-H	32–32.4
HFR2					
HCDR2	H52 A-G	H52 A-G		H52 A-G	60.1–60.4
HFR3	H82 A-E	H82 A-E		H72 A-E	84, 84.1–84.2
HCDR3	H100 A-T	H100 A-T		H100 A-T	111.1–112.1
HFR4					

In the latest statistical analysis of the numbering schemes and their definitions of CDRs, the AbM and IMGT schemes appear to have the most consistent definition of CDR residues compared to the traditionally used Chothia and Kabat systems (Zhu *et al.*, 2024). The Kabat scheme showed the most significant number of discrepancies, particularly in defining HCDR1 and HCDR2, as mentioned earlier. The Chothia scheme shares similar definitions of most loops with the Kabat scheme, though it includes modifications to HCDR1 and HCDR2. However, there have been several revisions to the Chothia scheme, particularly at the boundaries of the CDRs, leading to increased confusion. The ‘consensus’ Chothia numbering system, proposed by the Martin group, seems to offer a solution for standardizing CDR definitions, though their approach limits the range of residues included. The AbM scheme addresses the incorrect CDR definitions found in Kabat and Chothia, although it defines HCDR1 by combining the ranges from both Kabat and Chothia, making it the broadest definition of the HCDR1 loop among the four major numbering schemes we reviewed (Fig. 5). The IMGT scheme provides valuable insights from a gene sequence alignment perspective, extending its analysis to the entire immunoglobulin superfamily. Additionally, multiple platforms associated with these numbering schemes offer functions for a variety of applications. However, the definition of CDR2 and the inclusion of two residues in HCDR3 are important considerations, especially for mutagenesis projects.

While the CDRs play a crucial role in determining the antigen-binding specificity of an antibody, they do not encompass the entire paratope. Moreover, previous studies have indicated that a subset of residues within the CDRs are directly involved in interacting with the antigen. (Glaser *et al.*, 1992; Padlan, 1994; Chen *et al.*, 1999). The subset of residues that directly interact with the antigen was defined as selectivity determining region (SDR) by Padlan et al in 1995 (Padlan *et al.*, 1995). And SDR grafting has been successfully employed in antibody humanization to minimize potential immunogenicity while preserving or optimizing antigen-binding affinity (Muraro *et al.*, 1988; Tamura *et al.*, 2000; Kashmiri *et al.*, 2001, 2005). Previous studies have revealed that the residues involved in antibody–antigen interactions exhibit a certain level of conservation (Collis *et al.*, 2003; Ofran *et al.*, 2008). Thus, some of these residues are underrepresented when defining the CDRs solely based on sequence conservation (Ofran *et al.*, 2008). Lastly, it is widely recognized that the FRs of antibodies also contribute to antibody–antigen binding. Residues within the FRs can be classified into two categories based on their involvement in antigen binding (Sela-Culang *et al.*, 2013). Firstly, there are FR residues that have been demonstrated to directly participate in antibody–antigen interactions (Foote and Winter, 1992; Pospisil *et al.*, 1995; Holmes *et al.*, 2001). For instance, studies have shown that certain loops within the FR region 3 (HFR-3) contribute to approximately 1.3% of the antibody–antigen contacts (Capra and Kehoe, 1974; Raghunathan *et al.*, 2012). Secondly, FR residues primarily serve as structural support for the CDRs, facilitating their proper conformation and orientation (Holmes *et al.*, 2001; a). These FR residues contribute to the overall conformation of the binding site necessary for effective antigen binding (Haidar *et al.*, 2012).

While the traditional understanding was that CDRs were primarily responsible for antibody–antigen recognition, recent research has revealed that the story of antibody–antigen interactions goes beyond the boundaries of the CDRs. The emergence of computational technologies has provided us with more versatile tools to gain deeper insights into antibody–antigen interactions. These advanced techniques allow us to explore additional regions and factors involved in the recognition process, leading to a more comprehensive understanding of the complexities underlying antibody–antigen interactions.

## Current antibody numbering related tools

With the development of the various numbering schemes, an increasing number of tools have been developed throughout the past decade. Based on the rapidly increasing database, the tools for various functions have been developed to further assist the studies related to antibody research projects. Currently the three biggest antibody annotation toolkits are provided from the abYsis, IMGT and the OPIG (Oxford Protein Informatics Group) (Table 3).

**Table 3:** Computational Tools For Antibody Numbering. The tools and algorithms are categorized based on their associated platforms or current status: tools related to the Andrew Martin group, IMGT, SabDab, other platforms, and inactive tools. For each method, we provide the tool name, relevant references, and a web link to the software when available. For resources that are no longer actively maintained, we recommend reaching out to the original authors for further information.

Tool	Description	Link	Ref
Andrew C.R. Martin group related tools			
abYsis	A web-based antibody research platform that features an integrated database of antibody sequence and structure data, along with various tools for annotation, residue distribution analysis, and length distribution	http://www.abysis.org/	(Swindells *et al.*, 2017)
Abnum	An antibody numbering tool that assigns numbers according to several popular schemes, including Kabat, Chothia, and IMGT.	http://www.bioinf.org.uk/abs/abnum/	(Abhinandan and Martin, 2008)
AbYbank	A database of antibody sequences and structures, featuring multiple branch databases such as ABDB, Kabat, SACS, EMBLIg, and AbPDBseq.	http://www.abybank.org/	(Ferdous and Martin, 2018)
IMGT related tools			
IMGT Platform	Provides a standardized system based on conserved amino acid positions across immunoglobulins.	https://www.imgt.org	(Lefranc *et al.*, 2015)
IMGT/V-Quest	A web-based tool that analyzes and annotates the variable regions of immunoglobulin and T-cell receptor sequences, providing detailed information on their structure, germline origin, and diversity.	https://www.imgt.org/IMGT_vquest/input	(Giudicelli *et al.*, 2011)
IMGT/Allel-align	A web-based tool that aligns and compares immunoglobulin and T-cell receptor allele sequences, enabling the identification of sequence variations and the analysis of allele diversity.	https://www.imgt.org/Allele-Align/	(Frigoul and Lefranc, 2005)
IMGT/Collier-de-Perles	A web-based tool that visualizes the sequence and structural relationships between immunoglobulin and T-cell receptor variable regions, representing them as a ‘pearl necklace’ to highlight conserved and variable residues.	https://www.imgt.org/3Dstructure-DB/cgi/Collier-de-Perles.cgi	(Ruiz and Lefranc, 2002)
SAbDab related tools			
SAbDab	The Structural Antibody Database provides a comprehensive resource for antibody structures, including numbering based on several schemes.	https://opig.stats.ox.ac.uk/webapps/sabdab	(Dunbar *et al.*, 2014)
SAbPred	A suite of tools for antibody structure prediction and analysis, which includes functionality for antibody numbering.	https://opig.stats.ox.ac.uk/webapps/sabpred	(Dunbar *et al.*, 2016)
ANARCI	Provides numbering according to multiple schemes (Kabat, Chothia, Martin, IMGT, AHo) and includes functionality for T-cell receptor (TCR) identification.	https://opig.stats.ox.ac.uk/webapps/sabdab-sabpred/sabpred/anarci/	(Dunbar and Deane, 2016)
TAP	A tool for numbering antibody sequences, with a focus on high-throughput analysis and compatibility with other immunoinformatics tools.	https://opig.stats.ox.ac.uk/webapps/sabdab-sabpred/sabpred/tap	(Raybould *et al.*, 2018)
PEARS	A Python-based tool for antibody numbering and structural analysis, emphasizing speed and accuracy for large-scale antibody studies.	https://opig.stats.ox.ac.uk/webapps/sabdab-sabpred/sabpred/pears	(Leem *et al.*, 2016)
SCALOP	A structural comparison tool that assigns numbering based on structural alignment, often used in antibody engineering.	https://opig.stats.ox.ac.uk/webapps/sabdab-sabpred/sabpred/scalop	(Wong *et al.*, 2019b)
Other tools			
AbMAP	A high-throughput strategy that combines a phage-displayed peptide library with next-generation sequencing to map antibody epitopes in a single test​	–	(Qi *et al.*, 2021)
AbSRA	CDR definitions are based on sequence alignment and structural analysis, leveraging canonical structures and established antibody numbering schemes to define and annotate CDRs and key regions accurately.	http://cao.labshare.cn/AbRSA	(Li *et al.*, 2019)
BioPython	A widely used Python library that includes modules for antibody sequence analysis, including numbering according to various schemes.	https://biopython.org	(Cock *et al.*, 2009)
HMMER	Though primarily for sequence alignment, HMMER can be used for antibody numbering by aligning sequences to known antibody profiles.	http://hmmer.org/	(Eddy, 1998)
Homology Modeling Toolkit	An antibody modeling toolkit that includes numbering based on sequence and structural alignment.	–	(Krawczyk *et al.*, 2017)
IgAT	A computational software that includes functions for numbering, analyzing, and visualizing antibody sequences and structures.	www.uni-marburg.de/neonat/igat	(Rogosch *et al.*, 2012)
PyMOL Antibody Numbering	A plugin for the PyMOL molecular visualization tool that allows for numbering antibody sequences and structures according to several numbering schemes.	https://pymol.org/ ; https://pymolwiki.org/index.php/Annotate_v.	(by Schrödinger, 2024)
Inactive Tools			
AAAAA server (Inactive)	A tool dedicated to the AHo numbering scheme, ensuring consistency across different antibody frameworks by focusing on structural alignment.	https://plueckthun.bioc.uzh.ch/antibody/index.html	(Honegger and Plückthun, 2001b)
Paratome (Inactive)	Primarily a tool for defining ABRs; it also provides numbering based on structural alignment.	http://www.ofranlab.org/paratome/	(Kunik *et al.*, 2012a)
PvIg-Classify (Inactive)	Uses the IMGT numbering scheme and focuses on accurately classifying and numbering antibody sequences.	http://dunbrack2.fccc.edu/pyigclassify/	(Adolf-Bryfogle *et al.*, 2015)
Web Antibody Modeling (WAM) (Inactive)	An online tool for antibody structure prediction and numbering based on sequence data, integrating multiple numbering schemes.	http://antibody.bath.ac.uk /	(Whitelegg and Rees, 2000)

abYsis is a web-based antibody research system that includes an integrated database of antibody sequence and structure data developed and maintained by Andrew Martin group (Swindells *et al.*, 2017). It provides detailed information about the alignment, distribution, and annotation tools for the antibody sequences stored in their database (Swindells *et al.*, 2017). Furthermore, their website (http://www.bioinf.org.uk/) provides detailed intelligence about the current major numbering schemes and the differences among them which provides a detailed guidebook for antibody engineers to get to gain insight about the variable domain.

In addition to providing a new way to number and annotate antibodies, the IMGT information system provides more comprehensive tools for antibody annotation, alignment, 2D presentation and binding surface predictions (Ruiz and Lefranc, 2002; Monod *et al.*, 2004; Frigoul and Lefranc, 2005; Kushwaha *et al.*, 2024). The IMGT system can further benefit antibody engineers by gaining more information based on the sequence or structures of target antibodies to further facilitate immuno-therapeutic research projects.

SabDab, built by OPIG, is a database that compiles all available antibody structures from the PDB, offering standardized annotation and presentation. (Dunbar *et al.*, 2014). Based on the vast amount of structural information stored, SabDab developed various tools to help antibody engineers investigate antibody structures to assist in structural predictions, analysis and alignments (Dunbar *et al.*, 2016; Leem *et al.*, 2016, 2018; Wong *et al.*, 2019b).

Unfortunately, several servers have been inactive without sufficient maintenance, but they still have use as references. For example, the AAAAA tool focused on Aho numbering scheme which structurally aligns the antibodies to further identify several key conserved structures for future studies. (Honegger and Plückthun, 2001b). Paratomes provide an efficient way to identify the antigen binding region (ABR) on the antibodies which raises the question of which part of the CDRs are the determining residues for antibody binding (Kunik *et al.*, 2012a).

## Conclusions and future perspectives

Since the introduction of the first antibody numbering scheme in 1977, significant advances have been made to optimize, refine, and integrate these schemes into computational tools, supporting a wide range of antibody-related research initiatives. Among the most established numbering systems are Kabat, Chothia, AbM, and IMGT, which have become indispensable for defining CDRs and their boundaries solely based on sequence data. Despite the maturity of these technologies, it is noteworthy that only a few researchers have systematically compared the major current numbering schemes or highlighted the persistent inconsistencies among them. (Kunik *et al.*, 2012a; Zhu *et al.*, 2024). In the recent study, statistical analysis revealed that AbM and IMGT stand out as particularly robust frameworks among existing numbering systems (Zhu *et al.*, 2024). The AbM scheme builds upon and refines the Kabat and Chothia systems by correcting known errors and consolidating their features, thereby enhancing accuracy (Abhinandan and Martin, 2008). In contrast, the IMGT system introduces a novel perspective by tracing antibody sequences back to their germline genes, offering deeper immunological insights (Lefranc *et al.*, 1999). These innovations have led to the development of sophisticated tools for structural analysis and other functionalities, many of which are hosted on platforms such as the Martin group’s toolkit and the IMGT system. These platforms continue to expand the possibilities for antibody engineering and provide essential guidance for future research in the field. Additionally, alternative numbering schemes such as aHo, Gelfand, and Wolfguy, though less widely adopted compared to the four major systems, still contribute valuable perspectives on antibody variable domains. These schemes can serve as useful references for specific antibody engineering projects.

Reflecting on the current state of antibody numbering, it is evident that the primary goal of defining CDRs based on sequence data has largely been achieved. However, numerous questions remain regarding CDRs, framework residues, and their interactions. Firstly, it is widely recognized that not all CDR residues contribute equally to binding affinity, with some playing more critical roles than others (Padlan, 1994; Sela-Culang *et al.*, 2013). While the HCDR3 loop is the most dominant in binding, the distribution of other CDR loops involved in interactions appears to be more random and less predictable (Davies and Metzger, 1983; Rudolph *et al.*, 2006; Wong *et al.*, 2019c). This raises important questions about whether there are underlying patterns or if the binding interface behaves like a ‘pin-art’ display. Is it possible to predict and quantify the likelihood of specific residues playing a pivotal role in binding within future antibody numbering schemes? Secondly, framework residues, as their name implies, are essential for maintaining the overall structural integrity of antibodies (Foote and Winter, 1992). However, this raises the question: is structural stability the only function they serve? Recent research highlights the unique contributions of the HCDR4 loop to both affinity and stability, yet this loop is commonly classified as a framework in most current numbering schemes (Julian *et al.*, 2017). Furthermore, a highly conserved ‘SGSGSG’ motif in the variable light regions has been identified, but its specific functional role remains undefined (Zhu *et al.*, 2024). Framework residues can directly interact with the antigen and influence the conformation of CDRs, thus suggesting the need for a more comprehensive understanding of their role (Honegger and Plückthun, 2001c; Ewert *et al.*, 2004; Kunik *et al.*, 2012b). These findings underscore the importance of further evaluating framework contributions in order to advance our understanding and improve antibody engineering efforts. Lastly, as antibody numbering becomes increasingly integral to antibody engineering projects, it is essential to continue evaluating and refining current schemes.

As the number of antibodies being developed and added to the antibody database continues to grow, our understanding of their CDRs, and the intricacies of antibody–antigen interactions increases. With advances of bioinformatics tools, these developments have provided valuable insights for antibody engineering. In the future, further rapid progress in this field holds great potential for more precise and efficient antibody design. This will undoubtedly contribute to the advancement of antibody-based therapeutics and diagnostics, enhancing their impact in both research and clinical applications.

## CRediT Taxonomy

Zirui Zhu (Conceptualization [lead], Data curation [lead], Formal analysis [lead], Investigation [lead], Methodology [lead], Writing - original draft [lead], Writing - review & editing [lead]), Hossein Ashrafian (Conceptualization [supporting], Data curation [supporting], Formal analysis [supporting], Investigation [supporting], Writing - original draft [supporting], Writing - review & editing [supporting]), Navid Mohammadian Tabrizi (Conceptualization [supporting], Formal analysis [supporting], Investigation [supporting], Writing - original draft [supporting], Writing - review & editing [supporting]), Emily Matas (Data curation [supporting], Formal analysis [supporting], Investigation [supporting], Visualization [supporting], Writing - review & editing [supporting]), Louisa Girard (Data curation [supporting], Formal analysis [supporting], Investigation [supporting], Writing - review & editing [supporting]), Haowei Ma (Data curation [supporting], Formal analysis [supporting], Investigation [supporting], Writing - review & editing [supporting]), Edouard C. Nice (Formal analysis [supporting], Investigation [supporting], Project administration [lead], Supervision [lead], Writing - review & editing [supporting]).

## Notes

**Edited By**: Feng Ni
